# Evaluating the effect of bilateral transcutaneous auricular vagus nerve stimulation on motor function recovery after stroke: A multicenter, randomized controlled trial protocol

**DOI:** 10.1371/journal.pone.0352146

**Published:** 2026-07-17

**Authors:** Jia Wang, Xiaoyang Dong

**Affiliations:** Department of Rehabilitation Medicine, the First Affiliated Hospital, Jiangxi Medical College, Nanchang University, Nanchang 330006, Jiangxi, P.R. China; TIU: Tishk International University, IRAQ

## Abstract

**Introduction:**

Previous studies have demonstrated that vagus nerve stimulation (VNS) is an emerging approach for stroke rehabilitation and numerous studies have shown that VNS can promote motor function recovery and alleviate neurological deficits both in clinical and animal models. However, current clinical applications primarily involve invasive or unilateral stimulation with limited sample sizes and a focus mainly on upper limb function. Recent evidence indicates that bilateral transcutaneous auricular vagus nerve stimulation (BtaVNS) may offer superior signal conduction and therapeutic outcomes compared with unilateral left transcutaneous auricular vagus nerve stimulation (LtaVNS). To date, the efficacy and safety of BtaVNS for comprehensive limb motor rehabilitation after stroke remain underexplored. This study aims to address this gap by conducting a multicenter, randomized controlled trial evaluating BtaVNS in post-stroke patients with hemiplegic limb motor dysfunction.

**Methods and analysis:**

We will conduct a prospective, double-blind, multicenter randomized controlled trial involving 114 stroke patients diagnosed with hemiplegic limb motor dysfunction. Participants will be randomly assigned to one of three groups: a BtaVNS group, a LtaVNS group, or a control group. All groups will receive routine post-stroke rehabilitative therapy, while the intervention groups will additionally receive non-invasive taVNS: either bilaterally or on the left ear only. The taVNS parameters will be set at 20 Hz frequency, 300 µs pulse width, and individually adjusted intensity (0.5-1 mA), delivered for 30 minutes per session, twice daily, six days per week for four weeks. The co-primary outcomes include the Fugl-Meyer Assessment of Upper Extremity (FMA-UE) and Fugl-Meyer Assessment of Lower Extremity (FMA-LE). Secondary outcomes include the Wolf Motor Function Test (WMFT), Berg Balance Scale (BBS) and Modified Barthel Index (MBI). Exploratory outcomes include neuroimaging evaluations using functional near-infrared spectroscopy (fNIRS). Assessments will be conducted at baseline, after 2 weeks of treatment, after 4 weeks of treatment and at the 8-week follow-up. Safety and adverse events will be monitored throughout the study.

**Trial registration number:**

ChiCTR2400093825

## 1. Introduction

Stroke is a leading cause of long-term disability worldwide, with approximately 80% of survivors experiencing varying degrees of motor dysfunction, which significantly impairing quality of life and imposing socioeconomic burdens on families and healthcare systems [1,2]. The restoration of motor function after stroke, particularly in the hemiplegic limbs, remains a key challenge in neurorehabilitation.

Vagus nerve stimulation (VNS), a neuromodulation technique initially approved by the Food and Drug Administration (FDA) for treating intractable epilepsy and later for treatment-resistant depression, has shown promise in enhancing post-stroke recovery [3]. In recent years, preclinical studies have demonstrated that VNS can modulate neuroinflammatory responses, enhance neuroplasticity, and promote motor function recovery in stroke models [4]. Clinical trials have further reported that pairing VNS with motor rehabilitation significantly improves upper limb function in chronic ischemic stroke patients [5–7]. Notably, Dawson et al. conducted a pivotal multicenter randomized controlled trial showing that VNS combined with task-specific therapy led to meaningful improvements in motor outcomes compared with rehabilitation alone [6].

Among various VNS approaches, transcutaneous auricular vagus nerve stimulation (taVNS) has gained attention as a non-invasive alternative that stimulates the auricular branch of the vagus nerve. It offers a safer, more accessible modality compared with the traditional implanted VNS (iVNS), with comparable neuromodulatory effects [8]. Most existing clinical studies of taVNS for stroke rehabilitation have employed unilateral stimulation (typically on the left ear) and focused primarily on upper limb deficits. However, the existing literature suggests that bilateral transcutaneous auricular vagus nerve stimulation (BtaVNS) may provide greater sensory afferent input to brainstem pathways, thereby potentiating the therapeutic effects of VNS. Compared with unilateral left transcutaneous auricular vagus nerve stimulation (LtaVNS), BtaVNS appears to produce more pronounced neuromodulatory benefits [9,10].

Despite its potential, current evidence supporting taVNS in post-stroke rehabilitation is limited by small sample sizes, single-center designs, and a lack of emphasis on lower limb motor function. To address these limitations, we propose a multicenter, prospective, randomized controlled trial to evaluate the efficacy and safety of BtaVNS in improving upper and lower limb motor function in stroke patients. In addition to assessing clinical outcomes, we will employ neuroimaging techniques such as functional near-infrared spectroscopy (fNIRS) to explore the underlying neural mechanisms, offering a more comprehensive understanding of taVNS-induced motor recovery.

## 2. Methods and analysis

### 2.1. Study design

**Fig 1:**
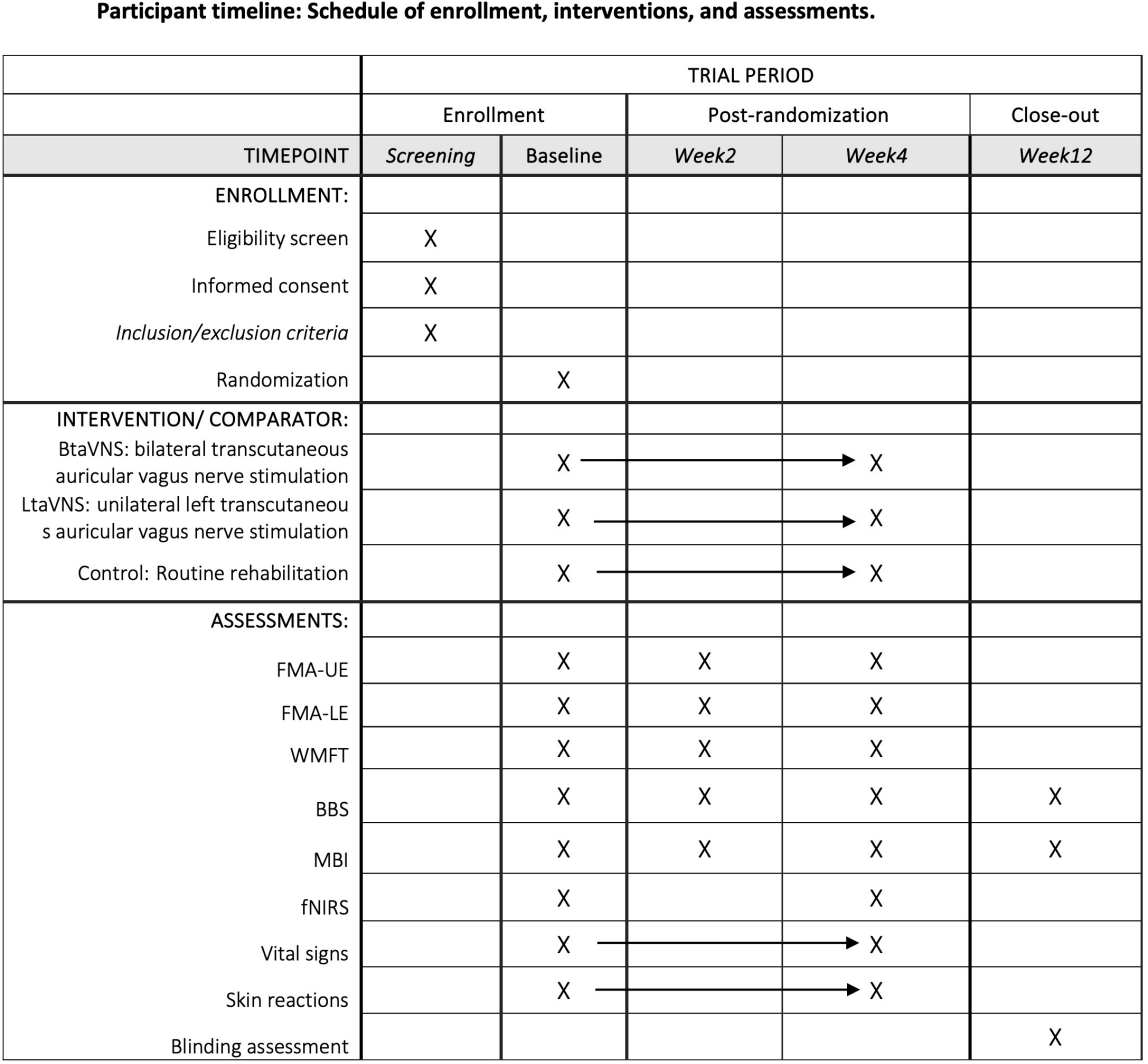
Participant timeline: Schedule of enrollment, interventions, and assessments. FMA-UE: Fugl–Meyer Assessment–upper extremity; FMA-LE: Fugl–Meyer Assessment–lower extremity; WMFT: Wolf Motor Function Test; BBS: Berg Balance Scale; MBI: Modified Barthel Index; LtaVNS: left-sided transcutaneous auricular vagus nerve stimulation; BtaVNS: bilateral transcutaneous auricular vagus nerve stimulation.

**Fig 2:**
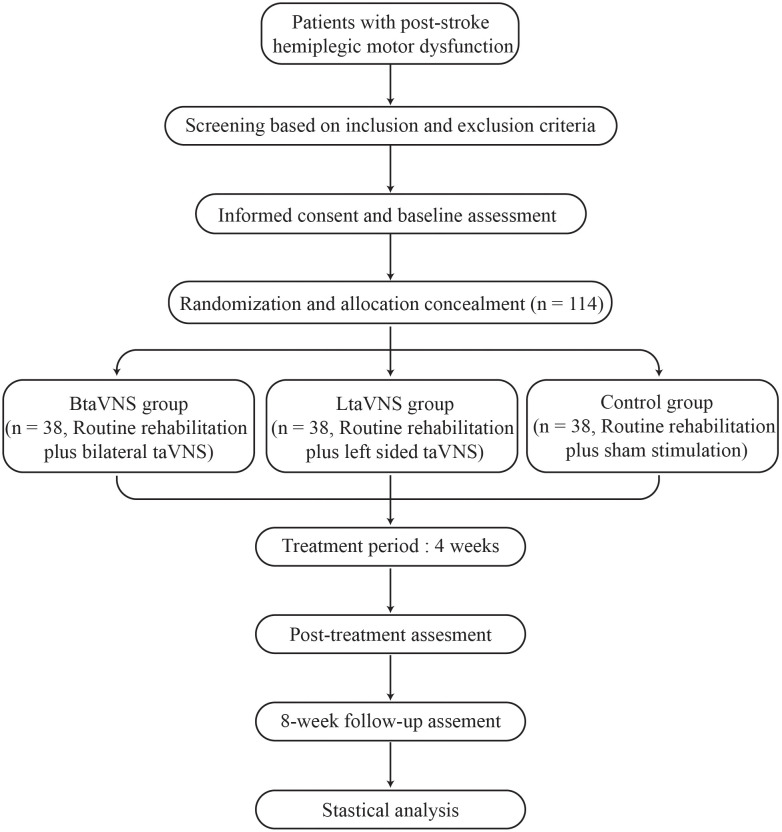
Study flowchart. LtaVNS: left transcutaneous auricular vagus nerve stimulation; BtaVNS: bilateral transcutaneous auricular vagus nerve stimulation.

This is a prospective, multicenter, double-blind, randomized controlled trial with three parallel arms. The trial will evaluate the efficacy and safety of BtaVNS compared with LtaVNS and routine rehabilitation in patients with post-stroke hemiplegic motor dysfunction. The study protocol has been approved by the Medical Research Ethics Committee of the First Affiliated Hospital of Nanchang University (IIT-2024–736) and registered in the Chinese Clinical Trial Registry (registration No. ChiCTR2400093825). This clinical trial will be conducted in accordance with the ethical principles of the Declaration of Helsinki. Prior to any study procedures, written informed consent will be obtained from all participants. As all participants will be adults (aged 18–65 years), consent from parents or guardians is not applicable. The trial will follow the SPIRIT guidelines for the design and reporting of randomized trials. Fig 1 shows the Participant timeline and Fig 2 illustrates the study flowchart.

### 2.2. Study status and timeline

This study is an ongoing randomized controlled trial. Participant recruitment commenced in January 2025 and is actively continuing. The recruitment period is projected to continue until December 31, 2027. As the intervention lasts for 4 weeks followed by an 8-week follow-up for each participant, the final data collection for the last enrolled participant is anticipated to be completed by February 29, 2028. Data cleaning, locking, and statistical analysis will proceed thereafter, with the primary study results expected to be available by June 2028.

### 2.3. Population

The study participants will be enrolled stroke patients with hemiplegic motor dysfunction, recruited from three major rehabilitation centers in Jiangxi Province, China: (1) The First Affiliated Hospital of Nanchang University; (2) The Affiliated Rehabilitation Hospital of Nanchang University; and (3) The First Affiliated Hospital of Gannan Medical University. All participants will be screened and enrolled by trained rehabilitation specialists according to strict inclusion and exclusion criteria to ensure participant safety and population homogeneity.

Inclusion criteria: (1) Aged 18–65 years; (2) Diagnosed with ischemic or hemorrhagic stroke in accordance with the 2021 Chinese Guidelines for Stroke Prevention and Treatment; (3) In the recovery phase of stroke (≥2 weeks post-onset), with stable vital signs and no ongoing disease progression; (4) Presenting with unilateral motor dysfunction (hemiplegia) affecting both the upper and lower limbs; (5) Capable of participating in standardized rehabilitation and taVNS treatment; (6) Able to provide signed informed consent.

Exclusion criteria: (1) Motor dysfunction resulting from causes other than stroke; (2) Severe cognitive impairment or psychiatric illness that would prevent compliance with treatment; (3) Unstable vital signs or a history of recurrent stroke at the time of screening; (4) Presence of implanted cardiac pacemakers or other contraindicated medical devices; (5) History of uncontrolled epilepsy, vasovagal syncope, or other conditions contraindicating vagus nerve stimulation.

Withdrawal criteria: (1) Sudden deterioration requiring transfer or termination of treatment; (2) Development of serious adverse events (e.g., recurrent stroke or life-threatening conditions); (3) Inability to tolerate taVNS due to persistent discomfort or pain; (4) Voluntary withdrawal by the patient or legal representative at any time.

### 2.4. Sample size

The required sample size was estimated using G*Power 3.1, based on effect sizes reported in previous randomized controlled trials evaluating vagus nerve stimulation paired with rehabilitation for post-stroke motor recovery. Specifically, the sample size calculation was informed by the VNS-REHAB trial conducted by Dawson et al., which demonstrated a clinically meaningful improvement in motor function with vagus nerve stimulation compared with rehabilitation alone, corresponding to an effect size of approximately Cohen’s d = 0.632 [6]. Although the VNS-REHAB trial employed invasive VNS, both invasive and transcutaneous auricular VNS engage overlapping vagal afferent pathways and share similar neuroplasticity mechanisms. In the absence of large-scale trials evaluating bilateral taVNS, this effect size was adopted as a conservative reference for estimating the anticipated treatment effect. The sample size was powered to detect the primary contrast of interest, namely the difference in motor function outcomes between the BtaVNS group and the control group. Therefore, the primary confirmatory comparison in this study is between the BtaVNS group and the control group. Comparisons between BtaVNS and LtaVNS, as well as between LtaVNS and the control group, are prespecified secondary analyses and are not specifically powered for confirmatory hypothesis testing. These additional comparisons are intended to provide further insight into the relative efficacy of bilateral versus unilateral stimulation. The effect size (Cohen’s d = 0.632) was converted to Cohen’s f = 0.316 for a three-group comparison. Assuming a two-sided type I error rate (α) of 0.05 and a statistical power (1 − β) of 80%, the minimum required sample size was calculated as 34 participants per group. To account for an anticipated dropout or loss to follow-up rate of approximately 10%, the target sample size was increased to 38 participants per group. With three parallel study arms (BtaVNS, LtaVNS, and control), the total planned sample size for this multicenter trial is 114 participants.

### 2.5. Randomization and allocation concealment

This study adopts a stratified block randomization approach. Participants will first be stratified by study center and then by stroke type (ischemic or hemorrhagic) to ensure balanced group distribution across clinical settings and stroke subtypes. Within each stratum, patients will be randomly assigned to one of three groups (BtaVNS group, LtaVNS group, and control group) in a 1:1:1 ratio using variable block sizes. The randomization sequence will be generated by an independent statistician who is not involved in the trial’s clinical implementation or data analysis. Group allocation results and randomization codes will be sealed in sequentially numbered opaque envelopes by the study coordinator and distributed to each participating site. After confirming eligibility and obtaining informed consent, site investigators will open the envelopes in sequence to determine group assignment, ensuring strict allocation concealment.

### 2.6. Interventional protocol

**Fig 3:**
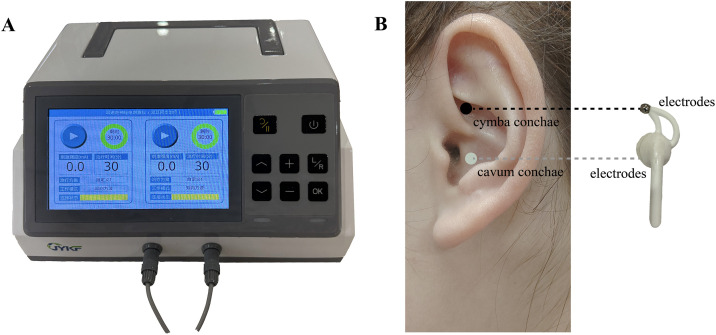
(A) BtaVNS device; (B) stimulation sites and electrodes. BtaVNS: bilateral transcutaneous auricular vagus nerve stimulation.

Following group allocation, all participants will receive standardized routine rehabilitation therapy and nursing care in accordance with current stroke rehabilitation guidelines. Participants in the intervention and control groups will additionally wear the taVNS stimulation device (JY-VNS-200, Jingyi Medical Technology Co., Ltd., Jiangxi, China, Fig 3A) to ensure blinding. The BtaVNS group will receive active stimulation simultaneously on both ears. Stimulation is delivered through paired metal electrodes mounted on a headphone-like apparatus, targeting the cymba conchae and cavum conchae regions (Fig 3B). Before each session, stimulation sites will be cleaned with alcohol to reduce impedance and ensure optimal conductivity. Stimulation parameters will be set as follows: square wave, 300 μs pulse width, 20 Hz frequency, 0.5–1 mA initial current intensity, for 30 minutes per session, twice daily, six days per week, for four weeks. The current may be adjusted upward in 0.5 mA increments based on patient tolerance. Behavioral cues will be monitored, and discomfort will be evaluated continuously.

The LtaVNS group will also wear the same device. Active stimulation will be applied only to the left ear, while the right ear will receive sham stimulation (0 mA current). All other parameters and procedures, including electrode placement and session duration, will be identical to those of the bilateral group to maintain blinding and treatment consistency. The control group will wear the taVNS device as well, but no electrical current (0 mA) will be delivered to either ear. The electrode placement, session duration, and monitoring procedures will mirror those of the intervention groups to ensure double-blind conditions. BtaVNS and LtaVNS will be performed at the same time before the start of the routine rehabilitation therapy in the morning and afternoon every day, under the operator’s continuous monitoring. To enhance safety, all devices include automatic impedance detection and alarm functions that are triggered when impedance exceeds 10 kΩ or if single-pulse energy exceeds 8 mJ, helping to prevent electrical burns or inadequate stimulation. The intervention protocol has been standardized across all participating centers to ensure reproducibility and facilitate its application in future clinical studies.

### 2.7. Blinding and unblinding

This study adopts a double-blind design. The A/B modes of the JY-VNS-200 device are visually and functionally identical, effectively blinding both the researchers and device operators to the stimulation status (active or sham). All participants are post-stroke patients undergoing routine rehabilitation and are unaware of their group allocation or the specific details of the stimulation intervention. We acknowledge the potential limitation that partial unblinding may occur due to sensory perception associated with active stimulation. To mitigate this risk, identical stimulation setups, electrode placement, and operational procedures are applied in all groups. In addition, outcome evaluators and data analysts are blinded to the treatment assignments to prevent subjective bias during assessment and statistical analysis. The randomization code and group assignment information are securely managed by an independent blinding supervisor who is not involved in any part of the study execution, data collection, or analysis. To assess blinding effectiveness, participants will be asked at the end of the intervention period to guess their group allocation (BtaVNS, LtaVNS, or control) and to indicate their confidence in this guess. The distribution of guesses will be analyzed descriptively to evaluate the success of blinding. Unblinding will only occur at the end of the trial, after the completion of all follow-up assessments. In cases of serious adverse events (e.g., cardiac arrest or other life-threatening complications) potentially related to taVNS, the sub-center may contact the blinding supervisor to request emergency unblinding for safety management and clinical decision making.

### 2.8. Data collection

After enrollment, baseline demographic and clinical information of participants in all three groups will be collected. This includes gender, age, stroke type (ischemic or hemorrhagic), time since stroke onset, lesion location (as indicated by CT or MRI), comorbidities (e.g., hypertension, diabetes), and initial scores on the Fugl-Meyer Assessment of Upper Extremity (FMA-UE) and Lower Extremity (FMA-LE), Wolf Motor Function Test (WMFT), Berg Balance Scale (BBS), and Modified Barthel Index (MBI).

**Fig 4:**
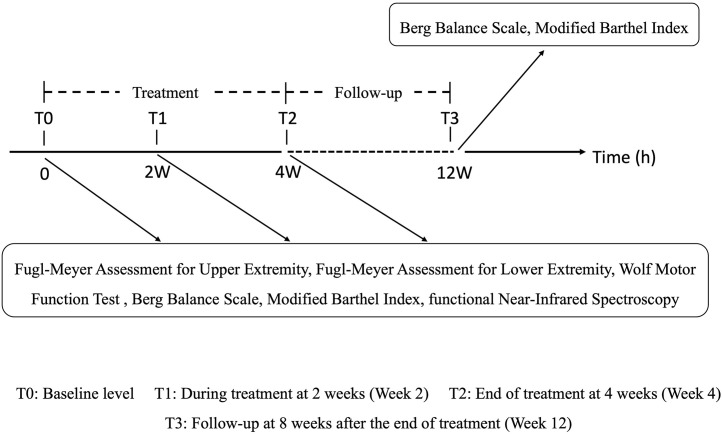
Evaluation flow diagram.

Patients will be reassessed at three main time points: after 2 weeks of treatment, after 4 weeks of treatment and at the 8-week follow-up. The co-primary outcomes are the change in FMA-UE and FMA-LE scores from baseline to the end of the 4-week intervention. Secondary outcomes include changes in WMFT, BBS and MBI scores at 2 weeks, at 4 weeks and at the 8-week follow-up. Follow-up assessments will be conducted either in person for inpatients or via structured interviews with the patient or caregivers for those discharged, using validated telephone versions of the outcome measures. The evaluation process during treatment and follow-up is shown in Fig 4.

To explore the potential neural mechanisms underlying BtaVNS for post-stroke motor dysfunction, this study will collect fNIRS data from participants before and after the 4-week intervention period. The Danyang Huichuang NirScan-6000A fNIRS equipment will be used for data acquisition. Before the examination, participants will be clearly informed of the study procedures and instructed to remain relaxed, avoid unnecessary head movements, and minimize swallowing or facial muscle contractions during the test to reduce artifacts and measurement errors. The regions of interest for this study include the bilateral primary motor cortex (M1), premotor cortex (PMC), supplementary motor area (SMA), and sensorimotor cortex (SMC), which are closely related to motor control and neuroplasticity. A 63-channel fiber-optic cap will be applied, consisting of 24 light sources and 24 detectors. During the assessment, near-infrared light emitted by the sources penetrates the scalp and skull, reaching the underlying cerebral cortex. The light is differentially absorbed by oxygenated hemoglobin (HbO) and deoxygenated hemoglobin (HbR), with the remaining light captured by the detectors. By monitoring the concentration changes of HbO and HbR within the channels, cortical hemodynamic responses and local neural activity associated with motor function can be indirectly evaluated.

Given the role of the vagus nerve in autonomic cardiovascular regulation, cardiovascular safety will be monitored throughout the trial using routine clinical monitoring methods, including heart rate, blood pressure, and ECG. These measures will enable the detection of clinically relevant hemodynamic changes, such as fluctuations in blood pressure and heart rate, as well as potential arrhythmias during taVNS. Heart rate will be measured using a fingertip pulse oximeter before and during the first stimulation session, while blood pressure and ECG will be monitored using a standard patient monitor (BenVision N12C, Shenzhen Mindray Bio-Medical Electronics Co., Ltd., China). In addition, skin safety at the stimulation sites will be documented throughout the trial, and any signs of irritation, including redness, blistering, or exudation, will be carefully recorded and evaluated.

All adverse events—whether related or unrelated to taVNS—will be systematically monitored and recorded throughout the study. These events may involve multiple systems, including neurological (e.g., epilepsy, hydrocephalus, intracranial infection), cardiovascular (e.g., arrhythmia, deep vein thrombosis, pulmonary embolism, cardiac arrest), respiratory (e.g., pneumonia, acute respiratory distress syndrome), gastrointestinal (e.g., gastrointestinal bleeding), and urological (e.g., urinary tract infection). In addition, the type, frequency, and duration of rehabilitation interventions received by each participant will be thoroughly documented to ensure consistency across groups and to allow for appropriate adjustments during statistical analysis.

### 2.9. Data management

All data collected from participants will be recorded in standardized case report forms (CRFs) by trained investigators at each participating center. Upon completion of the trial, the CRFs will be submitted to the coordinating center (The First Affiliated Hospital of Nanchang University). Data managers at the coordinating center will input and store the information anonymously using Excel spreadsheet to ensure data security and confidentiality. Access to the database will be restricted to authorized data management personnel only. Once data entry and verification are complete, the database will be locked and transferred to the study statistician for analysis in accordance with the pre-specified statistical analysis plan.

### 2.10. Statistical analysis

Continuous variables will be summarized as mean ± standard deviation, and categorical variables as counts and percentages. Normality of continuous variables will be assessed using the Shapiro–Wilk test. All efficacy analyses will follow the intention-to-treat (ITT) principle, including all randomized participants. The FMA-UE and FMA-LE will be defined as co-primary outcomes. To control the overall type I error rate associated with the two co-primary endpoints, a Bonferroni-adjusted significance level will be applied, with statistical significance defined as p < 0.025 for each primary outcome. The trial will be considered successful only if both co-primary endpoints (FMA-UE and FMA-LE) demonstrate statistically significant differences between groups at the adjusted significance level. Secondary and exploratory outcomes will be analyzed inferentially and interpreted with appropriate caution. Primary and secondary continuous outcomes measured repeatedly over time will be analyzed using linear mixed-effects models (LMMs). Fixed effects will include treatment group, time, and the group × time interaction, which will be used to evaluate differential changes over time between groups. Baseline outcome values, study center, age, sex, stroke type, and time since stroke onset will be included as covariates. Center effects will be modeled as fixed effects. When overall group effects are statistically significant, post hoc pairwise comparisons will be conducted with multiplicity adjustment using the Bonferroni method to control the family-wise error rate. For outcomes not meeting normality assumptions, appropriate data transformations or non-parametric methods will be applied. Categorical variables will be analyzed using Pearson’s chi-square test or Fisher’s exact test, as appropriate. Ordinal data will be analyzed using rank-based methods. Missing data are assumed to be missing at random (MAR). Linear mixed-effects models inherently accommodate missing outcome data under the MAR assumption. As a sensitivity analysis, multiple imputation will be performed for primary outcomes, and results will be compared with the primary analysis. Exploratory subgroup analyses by stroke type and lesion location will be conducted using interaction terms within the mixed-effects models. These analyses are considered hypothesis-generating and will be interpreted cautiously. A two-sided p value < 0.05 will be considered statistically significant for secondary and exploratory analyses unless otherwise specified. All statistical analyses will be performed using SPSS version 26.0.

## 3. Discussion

Despite the advancement of various rehabilitation strategies, functional recovery after stroke remains limited, partly due to the complex pathophysiology involving disrupted sensorimotor integration and maladaptive plasticity [11,12]. Vagus nerve stimulation, particularly its non-invasive variant taVNS has gained increasing attention as a promising neuromodulation strategy to augment post-stroke recovery [13,14]. Compared with implanted VNS, taVNS is safer, less expensive, and easier to administer. It targets the auricular branch of the vagus nerve (ABVN), predominantly located in the cymba and cavum conchae, thereby avoiding the cardiac risks associated with right cervical stimulation [15]. Neuroimaging studies demonstrate that taVNS activates brain regions such as the locus coeruleus and sensorimotor cortex via the nucleus tractus solitarius and thalamus [16,17].

Accumulating evidence suggests that taVNS paired with rehabilitative training can improve upper limb motor function in stroke survivors. A prior single-center RCT showed significant improvements in motor outcomes using unilateral LtaVNS, establishing Class II-level evidence for safety and efficacy [18]. Nonetheless, the study's limitations—including small sample size and lack of bilateral stimulation—prompted further investigation. Anatomical and neurophysiological studies indicate that BtaVNS may be more effective by activating both hemispheres and enhancing vagal afferent input to central motor networks. Importantly, research has shown that auricular stimulation activates primarily afferent fibers, reducing the likelihood of adverse cardiac effects. Accumulating evidence suggests that BtaVNS may provide superior activation of cortical networks and functional gains compared with unilateral stimulation [19,20]. Bilateral stimulation has been shown to promote interhemispheric balance and enhance motor cortex excitability in both hemispheres [15,21].

Several studies have confirmed taVNS’s ability to facilitate motor recovery when paired with rehabilitation tasks. Wang et al. and Badran et al. demonstrated that concurrent application of taVNS with task-oriented training or EMG-triggered movements leads to greater improvements in Fugl-Meyer Assessment (FMA) and cortical excitability [14,22]. Furthermore, Chang et al. showed that taVNS reduces spasticity and improves agonist-antagonist muscle coordination in chronic stroke patients [20]. From a mechanistic standpoint, taVNS influences neural plasticity through multiple pathways. Neurophysiological studies using EEG and motor-evoked potentials (MEPs) revealed enhanced cortical connectivity and excitability [23,24]. The fNIRS-based research has also shown increased activation in both sensorimotor and prefrontal cortices following taVNS [16]. Additionally, studies have suggested that taVNS facilitates neuroplasticity through enhanced functional connectivity [25], regulation of neuroinflammatory responses, and neurogenesis [12]. Regarding cardiovascular safety, we will monitor heart rate, blood pressure, and ECG during taVNS sessions, because the vagus nerve is involved in autonomic cardiovascular regulation. Although full heart rate variability parameters are not collected in this multicenter protocol, these physiological measures can still provide important safety information during stimulation. Most studies have shown no significant cardiovascular complications, further supporting the safety profile of BtaVNS [18,26].

Nevertheless, the present study has several limitations that should be acknowledged. Although the multicenter implementation enhances external validity by improving the generalizability of the findings across diverse clinical settings, the 8-week follow-up period was designed as an initial step to evaluate the short-to-medium-term efficacy and safety of BtaVNS combined with routine rehabilitation. While this timeframe allows assessment of early and short-term sustained post-intervention effects, it may be insufficient to fully capture the long-term maintenance of functional gains, particularly in patients with chronic stroke. In addition, due to variability in resources and clinical conditions across participating centers, comprehensive neuroimaging assessments and continuous blood pressure monitoring cannot be uniformly implemented. Furthermore, although the stimulation parameters were adopted from previously validated randomized controlled trials, the optimal taVNS protocol has not yet been definitively established [13,18]. Therefore, future research should prioritize pragmatic trials with extended follow-up durations, incorporate standardized multimodal monitoring, and explore home-based rehabilitation settings, personalized stimulation strategies, and dose–response relationships to further strengthen ecological validity and elucidate the long-term clinical benefits and mechanistic underpinnings of BtaVNS.

We acknowledge that the sample size estimation in the present protocol is primarily based on effect size data derived from a single pivotal randomized controlled trial. Although this study represents the most robust and well-powered clinical evidence currently available in this field, reliance on one reference study may limit the generalizability of the estimated effect size across diverse populations. Therefore, the results of this trial should be interpreted with appropriate caution, and future studies with larger samples and pooled effect estimates are warranted to further refine sample size assumptions. Additionally, another limitation of this study is the use of an upper age limit in the eligibility criteria. This restriction was implemented to reduce potential confounding from age-related comorbidities, to enhance participant safety, and to maintain population homogeneity in this protocol study. However, we acknowledge that excluding older individuals may limit the generalizability of the findings, particularly to elderly stroke populations who often present with greater clinical complexity. Future studies with broader age ranges will be necessary to evaluate the feasibility, safety, and effectiveness of taVNS in more heterogeneous and older patient populations.

In conclusion, taVNS is a promising, safe, and cost-effective neuromodulatory method to improve motor function after stroke. Our multicenter protocol using bilateral stimulation aims to provide high-level clinical evidence to guide the implementation of taVNS in standard stroke rehabilitation practice.

## Supporting information

S1 File SPIRIT 2025 checklist. (DOCX)

S2 File Study Protocol. (PDF)

S3 File Translation of Clinical Study Protocol. (PDF)
