# Supplementary material for: Evaluating the effect of bilateral transcutaneous auricular vagus nerve stimulation on motor function recovery after stroke: A multicenter, randomized controlled trial protocol
Source: PLoS One. 2026 Jul 17;21(7):e0352146. doi: 10.1371/journal.pone.0352146 (PMC13379013; doi:10.1371/journal.pone.0352146)
Supplement: S2 File — (PDF) [file pone.0352146.s002.pdf]

临床研究方案

项目名称：双耳迷走神经刺激促进脑卒中后运动功能重建：一项前瞻性、安全性、多中心、随机对照研究

版本号：第 1.0 版

版本日期：2024 年 08 月 31 日

研究单位：南昌大学第一附属医院、南昌大学附属康复医院、赣南医科大学第一附属医院

主要研究者：董晓阳

申办单位：南昌大学第一附属医院

保密声明

本研究方案包含的所有信息所有权归本项目的研究者所有，仅提供给伦理委员会和相关机构审阅。在未经主要研究者（PI）书面同意，严禁将任何信息告知与本研究无关的第三方。

主要研究者方案签字页

我已经认真阅读过本方案，同意方案中所有的内容，并且同意通过签名同意在本人所在的研究单位对此项目研究的实施进行监管，知晓需要获得所有参与研究受试者的知情同意书和相应的记录文件；确保临床研究按照研究方案、知情同意书、伦理委员会审查程序、赫尔辛基宣言等伦理原则及临床研究相关法律法规的要求开展研究。

主要研究者签名：____________ 签名日期：2024 年 11 月 14 日

方案摘要

**研究题目**	双耳迷走神经刺激促进脑卒中后运动功能重建：一项前瞻性、安全性、多中心、随机对照研究
**研究目的**	本项目旨在观察双耳迷走神经电刺激对脑卒中运动功能障碍重建的治疗效果以及安全性，并且通过 fNIRS 技术阐明其脑功能康复机制，从而为双耳迷走神经电刺激治疗卒中运动功能障碍的临床应用奠定理论基础。
**研究设计**	前瞻性、多中心、假性随机对照研究
**研究人群**	**纳入标准：**（1）年龄在 18 岁至 65 岁之间；（2）根据《中国脑卒中防治指南》，2021 年起经合格临床医生确诊为脑卒中（包括缺血性脑卒中和出血性脑卒中）；（3）脑卒中恢复期，病程大于 2 周，生命体征稳定，无疾病进展；（4）肢体运动功能障碍者，诊断为偏瘫。<br><br>**排除标准：**（1）由其他疾病导致的肢体运动功能障碍；（2）有精神系统疾病或认知功能障碍不能配合完成康复训练和耳迷走神经电刺激疗；（3）生命体征不稳定；（4）植入性心脏起搏器、未控制的癫痫，血管迷走神经性晕厥史，或有耳迷走神经电刺激的其他禁忌症等。
**干预措施**	（1）常规治疗组：在药物治疗的基础上，给与卒中偏瘫运动功能障碍常规康复治疗，包括运动治疗、作业疗法、物理因子治疗、普通针刺等综合康复治疗；（2）左侧耳迷走神经电刺激组：在常规治疗组的基础上，给与经典无创左侧耳迷走神经电刺激治疗。耳迷走神经电刺激治疗参数：左耳，频率 25Hz, 波宽 300us，电流 6 mA；（3）双耳迷走神经电刺激组:在常规治疗组的基础上，给与无创双耳迷走神经电刺激治疗。耳迷走神经电刺激治疗参数: 双耳，频率 25Hz, 波宽 300us，电流 6 mA。

**终点指标**	**主要研究终点的评价指标**：Fugl-Meyer 上肢(FMA-UE)和 Fugl-Meyer 下肢(FMA-LE)运动功能评定量表。<br><br>**次要研究终点的评价指标**：Wolf 运动功能评价量表(WMFT)、平衡评定与训练仪、Berg 平衡量表(BBS)、Holden 步行功能分级(FAC)以及改良 Barthel 指数(MBI)、功能性近红外光谱技术(fNIRS)、功能性磁共振(fMRI)检查。
**计划样本量及确定依据**	样本量采用 $N=2*[(Z_{\alpha/2}+Z_{\beta})\sigma/d]^2$ 和统计功率分析软件 G* power 3.1 确定。参考 Dawson et al 等研究实验中观察到的样本量(effect size, Cohen’s d=0.632)和 α=0.05，统计功率（1-β）等于 80 %，本研究每组的估计样本量应为 34。考虑到 10%的临床脱落率，每组的样本量调整为 38 人，三组共 114 人。
**统计分析**	统计分析将使用 R 软件进行。对于人口学及基线特征，若连续变量符合正态分布，将以均数 ± 标准差（$\bar{x}$ ± s）表示，并采用独立样本 t 检验进行组间比较；若连续变量不符合正态分布，则以中位数及四分位数间距（IQR）表示，并采用 Mann–Whitney U 检验进行分析。分类变量将根据样本容量和期望频数，使用卡方检验或 Fisher 精确检验进行比较。<br><br>对于主要与次要结局指标（如 FMA-UE、FMA-LE、WMFT、BBS 和 MBI 评分），将采用线性混合效应模型（LMM）处理重复测量数据，并通过 R 中的 “nlme” 包实现，从而评估随时间变化及组间差异，该模型可考虑同一受试者在多时间点数据间的相关性。事后亚组分析将探索 taVNS 在不同卒中类型（缺血性 vs. 出血性）及病灶部位下的差异性疗效。<br><br>对于安全性指标，将首先对心率变异性（HRV）及血压数据进行正态性检验。若符合正态分布，将使用配对样本 t 检验比较治疗前与治疗期间的数值；若不符合，则采用 Wilcoxon 符号秩检验。组间不良事件发生率的比较将使用卡方检验或 Fisher 精确检验。

	$p < 0.05$ 将被视为具有统计学意义。
**研究期限**	本项目预计 3 年完成（2025 年 1 月—2027 年 12 月）

缩略语列表

缩略语	定义
VNS	迷走神经电刺激
taVNS	耳迷走神经电刺激
FMA-UE	Fugl-Meyer 上肢运动量表
FMA-LE	Fugl-Meyer 下肢运动量表
BBS	Berg 平衡量表
FAC	Holden 步行功能分级
MBI	改良 Barthel 指数
fNIRS	功能性近红外光谱技术
fMRI	功能性磁共振

1. 研究背景

（1）脑卒中运动功能康复对推动健康中国建设具有重要的意义

据统计，我国脑卒中发病率为 150/10 万，排名世界第一，其中大约 80%的脑卒中患者存在偏瘫或肢体运动功能障碍[1-3]。卒中后肢体运动功能障碍限制了患者的生活半径和活动范围，严重降低了患者的生活质量和幸福感，给家庭和社会造成了沉重的经济负担和精神压力。因此如何有效地促进卒中患者偏瘫运动功能恢复，提高其生活质量，对推动"健康中国 2030 战略"建设具有重要的社会意义。

（2）脑卒中运动功能康复治疗方法与耳迷走神经电刺激

目前脑卒中后遗症期运动功能障碍的康复治疗方法主要包括运动治疗、物理因子治疗、作业疗法、传统中医治疗以及神经调控技术等[4, 5]。神经调控技术是近 20 年来神经科学领域发展较为迅猛的技术，在神经系统疾病治疗中具有广泛的应用。神经调控技术根据是否有创可分为无创神经调控技术和侵入性神经调控技术；根据刺激靶点位置可分为周围神经调控技术和中枢神经调控技术。然而由于部分神经调控技术部分因属于有创治疗、治疗周期较长以及疗效不明显，限制了其在卒中运动功能障碍康复临床应用和推广，因此探索新的神经调控治疗技术加速脑卒中运动功能重建进程具有重要意义。

迷走神经电刺激包括植入式迷走神经电刺激（Vagus Nerve Stimulation, VNS）和无创经耳迷走神经电刺激（Transcutaneous Auricular Vagus Nerve Stimulation，taVNS）。耳迷走神经电刺激属于无创周围神经调控技术，是通过刺激耳迷走神经分支进而激活相关脑区调控中枢神经活动达到治疗疾病效果（见示意图 1）。目前耳迷走神经电刺激主要应用于治疗难治性癫痫、复发性抑郁、脑损伤认知功能和脑损伤慢性意识障碍等疾病[6-8]。我们前期一项临床随机对照试验表明持续性左侧 taVNS 治疗可提高最小意识状态患者的觉醒水平，改善其脑电活动功能和诱发电位神经传导通路，且在治疗过程中未见与之相关的不良反应[9]。近年来，有文献逐步报道耳迷走神经电刺激应用于治疗卒中后运动功能障碍，引发了学术界广泛关注。

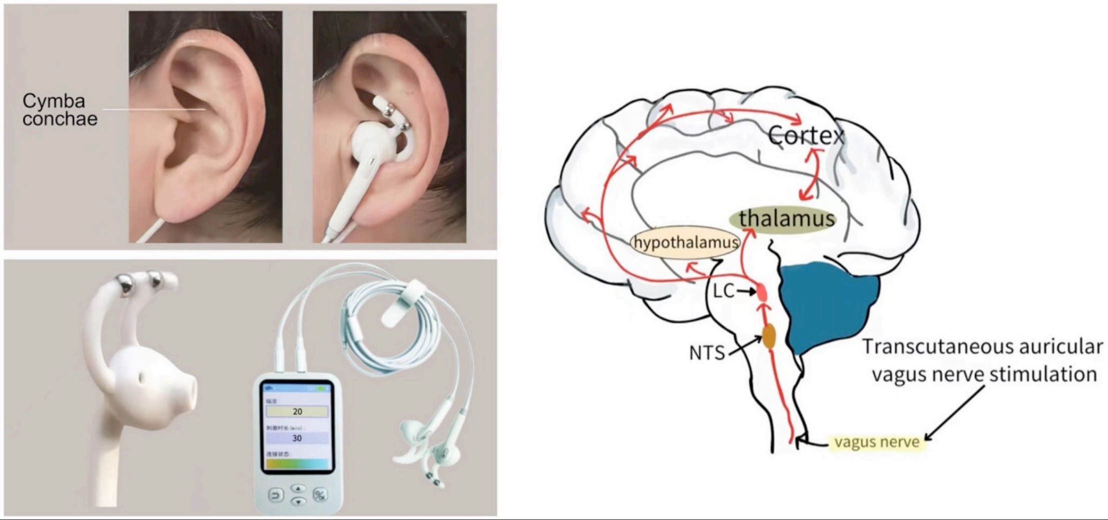

图 1 耳迷走神经电刺激治疗操作与机制示意图

（3）耳迷走神经电刺激重建卒中运动功能现状与不足

众多动物基础实验和临床试验均表明耳迷走神经电刺激能够促进脑卒中运动重建，改善其运动功能。2021年，Dawson等在Lancet、Neurorehabilitation and Neural Repair杂志上发表系列研究，采用随机双盲方法的临床试验表明植入式VNS联合运动功能训练对缺血性卒中患者上肢运动功能康复的效果明显优于假性VNS联合运动功能训练组[10, 11]。自此之后，广泛学者关注采用无创耳迷走神经电刺激在卒中后运动功能障碍恢复中的效果。Bashar等采用临床试验表明闭环耳迷走神经电刺激（Motor Activated Auricular Vagus Nerve Stimulation，MAAVN）能够提高卒中患者上肢Fugl-Meyer运动功能评分，改善其生活质量[12]。目前关于耳迷走神经电刺激治疗卒中运动功能障碍的机制主要包括[13-15]：减少卒中区域神

经元凋亡，减轻梗死面积，调节神经递质释放，抑制神经炎症因子相关通路激活，增强神经回路可塑性，改变血脑屏障通透性等。

然而当前迷走神经电刺激治疗卒中运动功能障碍仍存在一定的不足，**迷走神经电刺激治疗技术主要是有创植入或左侧单耳迷走神经电刺激治疗，观察对象为卒中后上肢运动功能障碍，且缺少多中心大样本的随机对照试验**。此外，文献表明激活**左右双耳迷走神经可增加对脑干神经传导通路的感觉输入，增强迷走神经电刺激治疗效果，效果优于单侧耳迷走神经电刺激**[16]。基于此，本项目将拟采用双耳迷走神经电刺激作为干预手段，卒中后上下肢运动功能障碍为研究对象，开展前瞻性、多中心、假性随机对照研究明确双耳迷走神经电刺激治疗卒中后上下肢运动功能障碍的临床疗效和安全性，阐明耳迷走神经电刺激治疗卒中运动功能障碍的脑功能康复机制。

2. 研究目的

2.1 **主要研究目的：**本项目旨在观察双耳迷走神经电刺激对脑卒中运动功能障碍重建的治疗效果，并且通过 fNIRS 和 fMRI 技术阐明其脑功能康复机制，从而为双耳迷走神经电刺激治疗卒中运动功能障碍的临床应用奠定理论基础。

2.2 次要研究目的：研究单耳迷走神经电刺激治疗卒中患者的安全性。

3. 研究概况

3.1 研究总体设计：本研究为一项前瞻性、安全性、多中心、随机对照研究。

3.2 研究人群

3.2.1 入选标准

（1）年龄在 18 岁至 65 岁之间；（2）根据《中国脑卒中防治指南》，2021 年起经合格临床医生确诊为脑卒中（包括缺血性脑卒中和出血性脑卒中）；（3）脑卒中恢复期，病程大于 2 周，生命体征稳定，无疾病进展；（4）肢体运动功能障碍者，诊断为偏瘫。

3.2.2 排除标准

（1）由其他疾病导致的肢体运动功能障碍；（2）有精神系统疾病或认知功能障碍不能配合完成康复训练和耳迷走神经电刺激治疗；（3）生命体征不稳定；（4）植入性心脏起搏器、未控制的癫痫，血管迷走神经性晕厥史，或有耳迷走神经电刺激的其他禁忌症等。

3.2.3 退出标准

（1）患者病情变化需要转科或患者不能够完成规范化治疗；（2）治疗过程中生命体征不稳定或再次发生卒中；（3）而迷走神经电刺激治疗不能耐受等。

3.3 研究分组及干预措施

本临床试验为验证双耳迷走神经电刺激治疗卒中运动功能障碍的临床疗效和安全性，以及与单侧左侧耳迷走神经电刺激治疗效果的差异性，共分成三组：

（1）**常规治疗组**：在药物治疗的基础上，给与卒中偏瘫运动功能障碍常规康复治疗，包括运动治疗、作业疗法、物理因子治疗、普通针刺等综合康复治疗。

（2）**左侧耳迷走神经电刺激组**：在常规治疗组的基础上，给与经典无创左侧耳迷走神经电刺激治疗。耳迷走神经电刺激治疗参数：左耳，频率 25Hz, 波宽 300us，电流 6 mA。

（3）**双耳迷走神经电刺激组**：在常规治疗组的基础上，给与无创双耳迷走神经电刺激治疗。耳迷走神经电刺激治疗参数：双耳，频率 25Hz, 波宽 300us，电流 6 mA。

耳迷走神经电刺激治疗方案：30min/次，2 次/日，6 天/周，4 周。

3.4 随机化分组

3.4.1 产生随机序列分配的方法

利用随机数字表进行随机分组。

3.4.2 随机分配的隐藏

中央随机

3.5 盲法及揭盲

采用双盲

3.6 研究程序

研究步骤主要包括临床试验随机入组，在基线水平（T0）、治疗 2 周时（T1）、4 周结束时（T2）以及治疗结束后 8 周（T3）进行随访采取进行相关指标采集，具体如下：

（1）随机入组：纳入符合标准的脑卒中运动功能障碍患者，将按照利用随机数字表将患者分别纳入常规治疗组、左耳迷走神经电刺激组和双耳迷走神经电刺激组。

（2）患者病史基本信息采集：纳入的研究对象将收集患者基本信息，包括年龄、性别、发病病程、卒中类型、教育背景、婚姻状况等。

（3）T0、T1 和 T2 时间点采集指标：Fugl-Meyer 上肢（FMA-UE）和 Fugl-Meyer 下肢（FMA-LE）运动功能评定量表、Wolf 运动功能评价量表（WMFT）、平衡评定与训练仪、Berg 平衡量表（BBS）、Holden 步行功能分级（FAC）以及改良 Barthel 指数（MBI）、功能性近红外光谱技术（fNIRS）。

（4）T2 随访时间点采集指标：通过电话或者门诊随访卒中患者治疗结束后 8 周 Berg 平衡量表（BBS）和 Barthel 指数（MBI）量表。

3.7 伴随治疗

药物治疗的基础上，给与卒中偏瘫运动功能障碍常规康复治疗。

3.8 终点评价指标

3.8.1 主要研究终点的评价指标

（1）Fugl-Meyer 上肢（FMA-UE）运动功能评定量表

（2）Fugl-Meyer 下肢（FMA-LE）运动功能评定量表

3.8.2 次要研究终点的评价指标：

（1）Wolf 运动功能评价量表（WMFT）

（2）平衡评定与训练仪、Berg 平衡量表（BBS）

（3）Holden 步行功能分级（FAC）

（4）改良 Barthel 指数（MBI）

（5）功能性近红外光谱技术（fNIRS）

（6）功能性磁共振（fMRI）检查

4. 安全性评价

4.1 不良事件

4.1.1 定义

不良事件（AE）：是在暴露于研究药物之后或期间的非预期的医疗状况或预先存在的医疗状况的恶化，无论其是否与研究药物有因果关系。非预期的医疗状况可以是症状（例如恶心、胸痛），体征（例如，心动过速、肝脏肿大）或检查的异常（例如实验室检查、ECG 检查）。正在研究的疾病发生的恶化及其相关症状或体征，如经研究者判断这种恶化可预期，则不被视为 AE。

严重不良事件（SAE）是指在研究期间发生的不良事件，其满足以下标准中的一个或多个：导致死亡、危及生命、需要住院治疗或延长目前的住院时间、导

致永久或显著的残疾/丧失工作能力或导致日常生活功能被严重破坏、导致先天性畸形或出生缺陷等。

4.1.2 严重程度

- 轻度：不适通常为短暂的，不影响日常生活及正常的活动

4.2 不良事件处理、随访和严重不良事件报告

研究者应向受试者详细说明，要求受试者如实反应采用干预治疗后的病情变化，医生避免诱导性提问，在观察疗效的同时，应密切关注不良事件，分析原因，作出判断，受试者研究过程中发生不良事件，应在病历中/病例报告表上详细记录不良事件的发生时间、症状、持续时间、处理措施和转归，并评价其与研究药物的关系；出现实验室检查异常者，须随访患者至检查结果恢复正常，或至用药前水平。发生严重不良事件应填写严重不良事件表，并在 24 小时内报告医院医学伦理委员会和 IIT 项目管理办公室。

4.3 不良事件相关性评定

研究者应对不良事件和研究药物可能存在关联性作出评估，不良事件与试验药物关系的判断标准如下：

①肯定无关:不良事件与试验药物的使用没有相关性。如：未使用试验药物。

②可能无关:没有证据显示事件的发生和试验药物之间存在因果关系。不良事件的发生更可能与其它因素有关，如：合并用药或伴随疾病。但是，二者之间的相关性无法被排除。

③可能有关:不良事件的发生与试验用药使用有合理的时间顺序，不良事件的发生可能由试验药物引起。不能排除是否可能由其它因素引起，如：合并用药或伴随疾病。没有进行撤药或者不清楚。

④肯定有关:不良事件的类型已被确认是药物已知的反应类型，而且不能用其它理由解释（如：合并用药和伴随疾病）。事件发生的时间强烈提示因果关系（如：撤药及再次服药后的反应）。

⑤ 无法评估:缺乏足够的信息来对事件和试验药物的因果关系做出判断。研究者可以根据后续的随访信息来改变她/他做的因果关系评估，并修改相应的 AE/SAE 报告。

5. 数据收集与管理

5.1 数据收集

电子数据记录

5.2 数据管理

采用 EXCEL 录入并整理数据、建立数据库。

6. 统计分析计划

6.1 样本量估算

样本量采用 $N=2*[(Z_{\alpha/2}+Z_{\beta})\sigma/d]^2$ 和统计功率分析软件 G* power 3.1 确定。参考 Dawson et al 等研究实验中观察到的样本量(effect size, Cohen’s d=0.632)和 α=0.05，统计功率（1-β）等于 80 %，本研究每组的估计样本量应为 34。考虑到 10%的临床脱落率，每组的样本量调整为 38 人，三组共 114 人。

参考文献：Dawson J, et al. Vagus nerve stimulation paired with rehabilitation for upper limb motor function after ischaemic stroke (VNS-REHAB): a randomised, blinded, pivotal, device trial. Lancet. (2021) 397:1545–53.

6.2 疗效分析及统计方法

统计分析将使用 R 软件进行。对于人口学及基线特征，若连续变量符合正态分布，将以均数 ± 标准差（$\bar{x} \pm s$）表示，并采用独立样本 t 检验进行组间比较；若连续变量不符合正态分布，则以中位数及四分位数间距（IQR）表示，并采用 Mann–Whitney U 检验进行分析。分类变量将根据样本容量和期望频数，使用卡方检验或 Fisher 精确检验进行比较。

对于主要与次要结局指标（如 FMA-UE、FMA-LE、WMFT、BBS 和 MBI 评分），将采用线性混合效应模型（LMM）处理重复测量数据，并通过 R 中的 “nlme” 包实现，从而评估随时间变化及组间差异，该模型可考虑同一受试者在多时间点数据间的相关性。事后亚组分析将探索 taVNS 在不同卒中类型（缺血性 vs. 出血性）及病灶部位下的差异性疗效。

对于安全性指标，将首先对心率变异性（HRV）及血压数据进行正态性检验。若符合正态分布，将使用配对样本 t 检验比较治疗前与治疗期间的数值；若不符合，则采用 Wilcoxon 符号秩检验。组间不良事件发生率的比较将使用卡方检验或 Fisher 精确检验。$p < 0.05$ 将被视为具有统计学意义。

。

6.3 分析人群

未依从研究方案的受试者予以剔除。

7. 研究相关伦理学

7.1 伦理委员会审查

本方案和书面知情同意书及与受试者直接相关的资料必须提交伦理委员会，获得伦理委员会书面批准后方可正式开展研究。研究者必须至少每年向伦理委员会提交研究年度报告。在研究中止和/或完成时，研究者必须书面通知伦理委员会；研究者必须及时向伦理委员会报告所有研究工作中发生的变化（如方案和/或知情同意书的修订），并且在未获得伦理委员会批准之前不得随意执行新的修改，除非是为了消除对受试者明显且直接的风险而做出的变更。在发生这类情况时，将通知伦理委员会。

7.2 知情同意

研究者必须向受试者或其法定代理人提供易于理解的并且经伦理委员会批准的知情同意书，并给与受试者或其法定代理人充分的时间考虑本项研究，在从受试者获得签署的书面知情同意书之前，受试者不得入组。 在受试者参与期间，将向受试者提供所有更新版本的知情同意书以及书面信息。知情同意书应作为临床试验的重要文档保留备查。

8. 保密措施

本项目研究的结果可能会在医学杂志上发表，但是我们会按照法律的要求为患者的信息保密，除非应相关法律要求，患者的个人信息不会被泄露。必要时，政府管理部门和医院伦理委员会及其有关人员可以按规定查阅患者的资料。

9. 质量保证措施

（1）**科学理论可行：**国内外文献和我们前期基础均表明单侧耳迷走神经电刺激可改善卒中后上肢运动功能障碍，且 fNIRS 发现耳迷走神经电刺激可激活卒中患者运动皮层。基于此，本项目拟开展双耳迷走神经电刺激治疗卒中偏瘫侧上肢和下肢运动功能的多中心临床试验，具有扎实的理论基础。

（2）**临床试验条件具备：**项目牵头单位南昌大学第一附属医院康复医学科是国家临床重点专科、江西省医学领先学科、江西省康复专科医联体发起单位，每年收治大量的脑卒中运动功能障碍患者，为本临床试验提供了充足的研究对象。项目分中心单位南昌大学附属康复医院和赣南医科大学第一附属医院为大型

综合性三级甲等医院，其康复医学科每年同样收治大量的卒中患者。此外，本科室已拥有双耳迷走神经电刺激治疗仪和 fNIRS 评估设备，具备完成临床试验相应的条件。

（3）**科研梯队合理：**本课题组长期致力于神经调控技术治疗脑损伤功能障碍康复治疗，项目组成员涉及到康复医学科、神经电生理学、影像科等多学科协作，科研梯队合理，分工明确，合作能力强，前期课题组相关人员具备完成课题的能力，在科研梯队上是可行。

（4）前期工作基础扎实：项目申请人在中国康复医学会副会长、中国康复医学会康复治疗专业委员会主任委员、中国康复医学会意识障碍康复专业委员会副主任委员冯珍教授的带领下，长期致力于脑损伤康复治疗方面的临床和基础研究工作。目前主持国家自然科学基金2项（青年1项、地区1项）、省级课题3项（省自然科学基金2项、省临床研究培育项目1项），参与多项国家自然科学基金项目，以第一作者或通讯作者发表论文15篇（其中SCI论文8篇），并且前期研究成果《周围神经电刺激昏迷促醒关键技术建立与应用》、《神经调控技术治疗慢性意识障碍关键技术创新与推广应用》分别获得2022年度江西省科学技术进步二等奖和2023年度中国康复医学会科学技术进步一等奖。此外，项目申请人及课题组成员熟练地掌握了临床研究的基本要求，具备了扎实的前期基础。

10. 研究项目的预期进度和完成日期

本项目预计 3 年完成，总体安排及进度如下：

<u>2025 年 1 月-2025 年 6 月</u>

临床试验前各项工作细化准备，脑卒中运动功能障碍患者和排除标准讲解，量表操作标准化培训，耳迷走神经电刺激治疗同质化培训，从而达到在三个临床分中心中达到同质化标准。此外，完成三个临床中心伦理申请和批复，在中国临床试验中心网站完成注册。

<u>2025 年 7 月-2026 年 6 月</u>

在南昌大学第一附属医院康复医学科首先进行卒中入组，行耳迷走神经电刺激治疗，关注其安全性，行 Fugl-Meyer 上肢（FMA-UE）和 Fugl-Meyer 下肢（FMA-LE）运动功能评定量表、Wolf 运动功能评价量表（WMFT）、平衡评定与训练仪、Berg 平衡量表（BBS）、Holden 步行功能分级（FAC）以及改良 Barthel

指数（MBI）、功能性近红外光谱技术（fNIRS）。

2026 年 07 月-2027 年 12 月

在南昌大学附属康复医院和赣南医科大学第一附属医院康复医学科进行卒中同步开展试验入组，行耳迷走神经电刺激治疗，关注其安全性，行 Fugl-Meyer 上肢（FMA-UE）和 Fugl-Meyer 下肢（FMA-LE）运动功能评定量表、Wolf 运动功能评价量表（WMFT）、平衡评定与训练仪、Berg 平衡量表（BBS）、Holden 步行功能分级（FAC）以及改良 Barthel 指数（MBI）、功能性近红外光谱技术（fNIRS）。

2027 年 12 月-2028 年 6 月

临床试验补充、数据收集和处理等统计实验数据，论文撰写，总结报告。

11. 研究成果的发表形式

（1）拟发表相关研究学术论文 1-2 篇，其中高影响力 SCI 期刊论文不少于 1 篇，并且并积极申报科研成果和专利 1-2 项。

（2）总结双耳迷走神经电刺激临床应用方案，建立临床应用示范单位≥3 家，依托《江西省康复专科医联体》平台将该技术在省内进行临床推广和应用。

12. 参考文献

[1] Saini V, Guada L, Yavagal D R. Global Epidemiology of Stroke and Access to Acute Ischemic Stroke Interventions[J]. Neurology, 2021,97(20 Suppl 2):S6-S16.

[2] Reinkensmeyer D J, Farrens A J, Kamper D G. Facilitating limb movement after stroke[J]. Nat Med, 2023,29(3):535-536.

[3] Le Danseur M. Stroke Rehabilitation[J]. Crit Care Nurs Clin North Am, 2020,32(1):97-108.

[4] Malik A N, Tariq H, Afridi A, et al. Technological advancements in stroke rehabilitation[J]. Journal of the Pakistan Medical Association, 2022,72(8):1672-1674.

[5] 张丽霞, 孟殿怀, 沈光宇, 等. 康复训练及针灸对偏瘫早期患者下肢运动功能恢复的作用[J]. 中国康复医学杂志, 2010,25(12):1179-1181.

[6] Dong X, Tang Y, Zhou Y, et al. Stimulation of vagus nerve for patients with disorders of consciousness: a systematic review[J]. Front Neurosci, 2023,17:1257378.

[7] Wang L, Wang Y, Wang Y, et al. Transcutaneous auricular vagus nerve stimulators: a review of past, present, and future devices[J]. Expert Rev Med Devices, 2022,19(1):43-61.

[8] Zhou Y F, Kang J W, Xiong Q, et al. Transauricular vagus nerve stimulation for patients with disorders of consciousness: A randomized controlled clinical trial[J]. Front Neurol, 2023,14:1133893.

[9] Zhou Y, Sun Y, He P, et al. The efficacy and safety of transcutaneous auricular vagus nerve stimulation for patients with minimally conscious state: a sham-controlled randomized double-blind clinical trial[J]. Front Neurosci, 2023,17:1323079.

[10] Dawson J, Engineer N D, Cramer S C, et al. Vagus Nerve Stimulation Paired With Rehabilitation

for Upper Limb Motor Impairment and Function After Chronic Ischemic Stroke: Subgroup Analysis of the Randomized, Blinded, Pivotal, VNS-REHAB Device Trial[J]. Neurorehabil Neural Repair, 2023,37(6):367-373.
[11] Dawson J, Liu C Y, Francisco G E, et al. Vagus nerve stimulation paired with rehabilitation for upper limb motor function after ischaemic stroke (VNS-REHAB): a randomised, blinded, pivotal, device trial[J]. Lancet, 2021,397(10284):1545-1553.
[12] Badran B W, Peng X, Baker-Vogel B, et al. Motor Activated Auricular Vagus Nerve Stimulation as a Potential Neuromodulation Approach for Post-Stroke Motor Rehabilitation: A Pilot Study[J]. Neurorehabil Neural Repair, 2023,37(6):374-383.
[13] Zhao X P, Zhao Y, Qin X Y, et al. Non-invasive Vagus Nerve Stimulation Protects Against Cerebral Ischemia/Reperfusion Injury and Promotes Microglial M2 Polarization Via Interleukin-17A Inhibition[J]. J Mol Neurosci, 2019,67(2):217-226.
[14] Ay I, Lu J, Ay H, et al. Vagus nerve stimulation reduces infarct size in rat focal cerebral ischemia[J]. Neurosci Lett, 2009,459(3):147-151.
[15] Bowles S, Hickman J, Peng X, et al. Vagus nerve stimulation drives selective circuit modulation through cholinergic reinforcement[J]. Neuron, 2022,110(17):2867-2885.
[16] Wang Y, Li S Y, Wang D, et al. Transcutaneous Auricular Vagus Nerve Stimulation: From Concept to Application[J]. Neurosci Bull, 2021,37(6):853-862.
