# Supplementary material for: Evaluating the effect of bilateral transcutaneous auricular vagus nerve stimulation on motor function recovery after stroke: A multicenter, randomized controlled trial protocol
Source: PLoS One. 2026 Jul 17;21(7):e0352146. doi: 10.1371/journal.pone.0352146 (PMC13379013; doi:10.1371/journal.pone.0352146)
Supplement: S3 File — (PDF) [file pone.0352146.s003.pdf]

Clinical Study Protocol

Project Title: Bilateral Auricular Vagus Nerve Stimulation for Promoting Motor Function Reconstruction after Stroke: A Prospective, Safety, Multicenter, Randomized Controlled Study

Version Number: 1.0

Version Date: August 31, 2024

Research Units: The First Affiliated Hospital of Nanchang University, The Rehabilitation Hospital Affiliated to Nanchang University, The First Affiliated Hospital of Gannan Medical University

Principal Investigator: Dong Xiaoyang

Sponsor: The First Affiliated Hospital of Nanchang University

Confidentiality Statement

All information contained in this study protocol is the property of the investigators of this project and is provided solely for review by the Ethics Committee and relevant regulatory bodies. Disclosure of any information to third parties unrelated to this study is strictly prohibited without the prior written consent of the Principal Investigator (PI).

[Principal Investigator Protocol Signature Page]{.underline}

I have carefully read this protocol, agree to all its contents, and consent through my signature to supervise the implementation of this project at my research site. I am aware of the need to obtain informed consent forms and corresponding documentation from all participating subjects; and ensure that the clinical study is conducted in accordance with the study protocol, informed consent form, Ethics Committee review procedures, the ethical principles of the Declaration of Helsinki, and relevant clinical research laws and regulations.

Principal Investigator Signature: ________________ Signature Date: [November 14, 2024]{.underline}

Protocol Synopsis

**Study Title**	Bilateral Auricular Vagus Nerve Stimulation for Promoting Motor Function Reconstruction after Stroke: A Prospective, Safety, Multicenter, Randomized Controlled Study

**Study Objective**	This project aims to observe the therapeutic effect and safety of bilateral auricular vagus nerve electrical stimulation on motor dysfunction reconstruction after stroke, and to elucidate its brain function rehabilitation mechanism using fNIRS technology, thereby laying a theoretical foundation for the clinical application of bilateral auricular vagus nerve electrical stimulation in treating post-stroke motor dysfunction.
**Study Design**	Prospective, multicenter, sham-randomized controlled study
**Study Population**	**Inclusion Criteria:** (1) Age between 18 and 65 years; (2) Diagnosed with stroke (including ischemic stroke and hemorrhagic stroke) by a qualified clinician according to the "Chinese Stroke Prevention and Treatment Guidelines" from 2021 onwards; (3) Stroke recovery period , stable vital signs, no disease progression; (4) Presence of limb motor dysfunction, diagnosed with hemiplegia. **Exclusion Criteria:** (1) Limb motor dysfunction caused by other diseases; (2) Presence of psychiatric system diseases or cognitive dysfunction preventing cooperation with rehabilitation training and taVNS therapy; (3) Unstable vital signs; (4) Implanted cardiac pacemaker, uncontrolled epilepsy, history of vasovagal syncope, or other contraindications for taVNS.
**Interventions**	(1) **Conventional Treatment Group:** Based on drug therapy, provide conventional rehabilitation treatment for stroke hemiplegic motor dysfunction, including comprehensive rehabilitation such as exercise therapy, occupational therapy, physical agent therapy, conventional acupuncture, etc. (2) **Left-sided taVNS Group:** Based on the conventional treatment group, provide classic non-invasive left-sided auricular vagus nerve electrical stimulation. taVNS parameters: left ear, frequency 25Hz, pulse width 300us, current 6 mA. (3) **Bilateral taVNS Group:** Based on the conventional treatment group, provide non-invasive bilateral auricular vagus nerve electrical stimulation. taVNS parameters: both ears, frequency 25Hz, pulse width 300us, current 6 mA.
**Endpoint Measures**	**Primary Endpoint Evaluation Indicators:** Fugl-Meyer Assessment for Upper Extremity (FMA-UE) and Fugl-Meyer Assessment for Lower Extremity (FMA-LE). **Secondary Endpoint Evaluation Indicators:** Wolf Motor Function Test (WMFT), Balance evaluation and training system, Berg Balance Scale (BBS), Holden Functional Ambulation Classification (FAC), Modified Barthel Index (MBI), functional Near-Infrared Spectroscopy (fNIRS), functional Magnetic Resonance Imaging (fMRI).
**Planned Sample Size & Justification**	Sample size was determined using N=2*\[(Zα/2+Zβ)σ/d\]^2^ and statistical power analysis software G*Power 3.1. Referring to the effect size observed in studies like Dawson et al. (Cohen's d=0.632), with α=0.05 and statistical power (1-β) equal to 80%, the estimated sample size per group should be 34. Considering a 10% clinical dropout rate, the sample size per group was adjusted to 38, totaling 114 participants across three groups.
**Statistical Analysis**	Statistical analysis will be performed using R software. For demographic and baseline characteristics, continuous variables conforming to normal distribution will be expressed as mean ± standard deviation ($\bar{x}$ ± s) and compared between groups using independent samples t-test; if not normally distributed, they will be expressed as median and interquartile range (IQR) and analyzed using Mann-Whitney U test. Categorical variables will be compared using Chi-square test or Fisher's exact test based on sample size and expected frequencies. For primary and secondary outcome measures (e.g., FMA-UE, FMA-LE, WMFT, BBS, MBI scores), Linear Mixed Effects Models (LMM) will be used to

handle repeated measures data, implemented via the "nlme" package in R, to assess changes over time and differences between groups, accounting for correlations within subjects across multiple time points. Post-hoc subgroup analyses will explore differential efficacy of taVNS across different stroke types (ischemic vs. hemorrhagic) and lesion locations.
 For safety indicators, normality tests will first be performed on Heart Rate Variability (HRV) and blood pressure data. If normally distributed, paired sample t-tests will be used to compare values before and during treatment; if not, Wilcoxon signed-rank test will be used. Comparison of adverse event incidence rates between groups will use Chi-square test or Fisher's exact test. $p < 0.05$ will be considered statistically significant. |
| **Study Duration** | This project is expected to be completed in 3 years (January 2025 --- December 2027). |

Abbreviations List

**Abbreviation**	**Definition**
VNS	Vagus Nerve Stimulation
taVNS	Transcutaneous Auricular Vagus Nerve Stimulation
FMA-UE	Fugl-Meyer Assessment for Upper Extremity
FMA-LE	Fugl-Meyer Assessment for Lower Extremity
BBS	Berg Balance Scale
FAC	Functional Ambulation Classification
MBI	Modified Barthel Index
fNIRS	functional Near-Infrared Spectroscopy
fMRI	functional Magnetic Resonance Imaging

1. Research Background

(1) Significance of Motor Function Rehabilitation after Stroke for Promoting Healthy China Construction

According to statistics, the incidence of stroke in China is 150/100,000, ranking first in the world, and approximately 80% of stroke patients experience hemiplegia or limb motor dysfunction[1-3]. Post-stroke limb motor dysfunction limits patients' life radius and range of activities, severely reduces their quality of life and sense of well-being, and places a heavy economic burden and mental pressure on families and society. Therefore, effectively promoting the recovery of motor function in stroke patients with hemiplegia and improving their quality of life hold significant social importance for advancing the "Healthy China 2030" strategy.

(2) Motor Function Rehabilitation Methods after Stroke and Transcutaneous Auricular Vagus Nerve Stimulation

Current rehabilitation treatments for motor dysfunction in the sequela phase of stroke mainly

include exercise therapy, physical agent therapy, occupational therapy, traditional Chinese medicine treatments, and neuromodulation techniques^[4, 5]^. Neuromodulation technology has developed rapidly in the field of neuroscience over the past 20 years and has wide applications in the treatment of nervous system diseases. Neuromodulation techniques can be classified as non-invasive or invasive based on whether they involve penetration, and as peripheral or central neuromodulation techniques based on the stimulation target site. However, some neuromodulation techniques are limited in their clinical application and promotion for post-stroke motor dysfunction rehabilitation due to factors such as being invasive, having long treatment cycles, or exhibiting unclear efficacy. Therefore, exploring new neuromodulation therapies to accelerate the process of motor function reconstruction after stroke is of great importance.

Vagus nerve stimulation includes implanted vagus nerve stimulation (VNS) and non-invasive transcutaneous auricular vagus nerve stimulation (taVNS). taVNS is a non-invasive peripheral neuromodulation technique that stimulates the auricular branch of the vagus nerve to activate related brain areas and regulate central nervous system activity to achieve therapeutic effects (see Schematic Diagram 1). Currently, taVNS is primarily used to treat diseases such as refractory epilepsy, recurrent depression, cognitive function in brain injury, and chronic disorders of consciousness after brain injury^[6-8]^. Our previous randomized controlled trial showed that continuous left-sided taVNS treatment could improve the arousal level of patients in a minimally conscious state, enhance their brain electrical activity function and evoked potential neural conduction pathways, with no related adverse reactions observed during treatment^[9]^. In recent years, literature has gradually reported the application of taVNS in treating post-stroke motor dysfunction, attracting widespread academic attention.

Figure 1 Schematic Diagram of taVNS Treatment Operation and Mechanism

(3) Current Status and Shortcomings of taVNS in Reconstructing Stroke Motor Function

Numerous basic animal experiments and clinical trials have shown that taVNS can promote motor reconstruction after stroke and improve motor function. In 2021, Dawson et al. published a series of studies in *Lancet* and *Neurorehabilitation and Neural Repair*, using randomized double-blind clinical trials, demonstrating that implanted VNS combined with motor function training was significantly more effective than sham VNS combined with motor function training for upper limb motor function rehabilitation in ischemic stroke patients^[10, 11]^. Since then, extensive attention has been paid to the effect of non-invasive taVNS on the recovery of motor dysfunction after stroke. Bashar et al. used a clinical trial to show that closed-loop taVNS (Motor Activated Auricular Vagus Nerve Stimulation, MAAVN) could improve the upper limb Fugl-Meyer motor function score and quality of life in stroke patients^[12]^. The current mechanisms of taVNS for treating post-stroke motor dysfunction mainly include^[13-15]^: reducing neuronal apoptosis in the stroke area, reducing infarct volume, regulating neurotransmitter release, inhibiting the activation of neuroinflammatory factor-related pathways, enhancing neural circuit plasticity, and altering blood-brain barrier permeability.

However, there are still some shortcomings in VNS for treating post-stroke motor dysfunction. **VNS techniques are mainly invasive implants or left-sided unilateral taVNS, the observation subjects are mainly upper limb motor dysfunction after stroke, and there is a lack of large-sample, multicenter randomized controlled trials.** Furthermore, literature suggests that activating **bilateral auricular vagus nerves can increase sensory input to brainstem neural pathways, enhancing the therapeutic effect of VNS, with effects superior to unilateral taVNS**^[16]^. Based on this, this project intends to use bilateral taVNS as the intervention, target upper and lower limb motor dysfunction after stroke, and conduct a prospective, multicenter, sham-randomized controlled study to clarify the clinical efficacy and safety of bilateral taVNS for treating upper and lower limb motor dysfunction after stroke, and to elucidate the brain function rehabilitation mechanism of taVNS in treating post-stroke motor dysfunction.

2. Study Objectives

2.1 **Primary Research Objective:** This project aims to observe the therapeutic effect of bilateral taVNS on motor dysfunction reconstruction after stroke, and to elucidate its brain function rehabilitation mechanism using fNIRS and fMRI technology, thereby laying a theoretical foundation for the clinical application of bilateral taVNS in treating post-stroke motor dysfunction.

2.2 **Secondary Research Objective:** To study the safety of unilateral taVNS in treating stroke patients.

3. Study Overview

3.1 Overall Study Design: This is a prospective, safety, multicenter, randomized controlled study.

3.2 Study Population

3.2.1 Inclusion Criteria
(1) Age between 18 and 65 years;
(2) Diagnosed with stroke (including ischemic stroke and hemorrhagic stroke) by a qualified clinician according to the "Chinese Stroke Prevention and Treatment Guidelines" from 2021 onwards;
(3) Stroke recovery period, stable vital signs, no disease progression;
(4) Presence of limb motor dysfunction, diagnosed with hemiplegia.

3.2.2 Exclusion Criteria
(1) Limb motor dysfunction caused by other diseases;
(2) Presence of psychiatric system diseases or cognitive dysfunction preventing cooperation with rehabilitation training and taVNS therapy;

(3) Unstable vital signs;
(4) Implanted cardiac pacemaker, uncontrolled epilepsy, history of vasovagal syncope, or other contraindications for taVNS.

3.2.3 Withdrawal Criteria
(1) Patient's condition changes requiring transfer to another department or inability to complete standardized treatment;
(2) Unstable vital signs during treatment or recurrence of stroke;
(3) Intolerance to taVNS treatment, etc.

3.3 Study Groups and Interventions
To verify the clinical efficacy and safety of bilateral taVNS for stroke motor dysfunction, and its difference compared to unilateral left-sided taVNS, this clinical trial is divided into three groups:
(1) **Conventional Treatment Group:** Based on drug therapy, provide conventional rehabilitation treatment for stroke hemiplegic motor dysfunction, including comprehensive rehabilitation such as exercise therapy, occupational therapy, physical agent therapy, conventional acupuncture, etc.
(2) **Left-sided taVNS Group:** Based on the conventional treatment group, provide classic non-invasive left-sided auricular vagus nerve electrical stimulation. taVNS parameters: left ear, frequency 25Hz, pulse width 300us, current 6 mA.
(3) **Bilateral taVNS Group:** Based on the conventional treatment group, provide non-invasive bilateral auricular vagus nerve electrical stimulation. taVNS parameters: both ears, frequency 25Hz, pulse width 300us, current 6 mA.

taVNS Treatment Regimen: 30min/session, 2 sessions/day, 6 days/week, for 4 weeks.

3.4 Randomization

3.4.1 Method for Generating Random Allocation Sequence
> Use a random number table for randomization.

3.4.2 Allocation Concealment
Central randomization.

3.5 Blinding and Unblinding
Double-blind design will be used.

3.6 Study Procedures
Study steps mainly include clinical trial randomization and grouping, follow-up and data collection at baseline (T0), at 2 weeks of treatment (T1), at the end of 4 weeks (T2), and at 8 weeks after treatment completion (T3), as detailed below:
(1) **Randomization and Grouping:** Enroll eligible stroke patients with motor dysfunction and assign them to the Conventional Treatment Group, Left-sided taVNS Group, or Bilateral

taVNS Group using a random number table.
(2) **Collection of Basic Patient History Information:** Collect basic information for enrolled subjects, including age, gender, time since onset, stroke type, education level, marital status, etc.
(3) **Data Collection at T0, T1, T2 time points:** Fugl-Meyer Assessment for Upper Extremity (FMA-UE) and Lower Extremity (FMA-LE), Wolf Motor Function Test (WMFT), Balance evaluation and training system, Berg Balance Scale (BBS), Holden Functional Ambulation Classification (FAC), Modified Barthel Index (MBI), functional Near-Infrared Spectroscopy (fNIRS).
(4) **Data Collection at T3 follow-up time point:** Follow up via phone or outpatient visit to collect Berg Balance Scale (BBS) and Modified Barthel Index (MBI) scores at 8 weeks after treatment completion.

3.7 Concomitant Treatment
Based on drug therapy, provide conventional rehabilitation treatment for stroke hemiplegic motor dysfunction.

3.8 Endpoint Evaluation Measures

3.8.1 Primary Endpoint Evaluation Measures
> (1) Fugl-Meyer Assessment for Upper Extremity (FMA-UE)
> (2) Fugl-Meyer Assessment for Lower Extremity (FMA-LE)

3.8.2 Secondary Endpoint Evaluation Measures:
> (1) Wolf Motor Function Test (WMFT)
> (2) Balance evaluation and training system, Berg Balance Scale (BBS)
> (3) Holden Functional Ambulation Classification (FAC)
> (4) Modified Barthel Index (MBI)
> (5) functional Near-Infrared Spectroscopy (fNIRS)
> (6) functional Magnetic Resonance Imaging (fMRI)

4. Safety Evaluation

4.1 Adverse Events

4.1.1 Definition
> **Adverse Event (AE):** Any untoward medical occurrence or worsening of a pre-existing medical condition in a subject temporally associated with the use of the investigational intervention, regardless of whether it is considered causally related. An untoward medical occurrence can be a symptom (e.g., nausea, chest pain), sign (e.g., tachycardia, hepatomegaly), or abnormal finding (e.g., laboratory test, ECG). Worsening of the disease under investigation and its associated symptoms or signs, if judged by the investigator to be expected, is not considered an AE.
>

> **Serious Adverse Event (SAE):** An adverse event occurring during the study that meets one or more of the following criteria: results in death, is life-threatening, requires inpatient hospitalization or prolongation of existing hospitalization, results in persistent or significant disability/incapacity, or severely disrupts activities of daily living, results in a congenital anomaly/birth defect, etc.

4.1.2 Severity
- Mild: Discomfort is usually transient, not interfering with daily life and normal activities.

4.2 Adverse Event Handling, Follow-up, and Serious Adverse Event Reporting
> The investigator shall explain in detail to the subject, requiring the subject to truthfully report any changes in condition after receiving the intervention treatment. Physicians should avoid leading questions. While observing efficacy, close attention should be paid to adverse events, analyzing causes and making judgments. For any AE occurring during the study, details such as time of onset, symptoms, duration, measures taken, and outcome should be recorded in the medical record/Case Report Form, and its relationship to the investigational intervention should be assessed; for those with abnormal laboratory findings, follow up until results return to normal or to pre-treatment levels. In case of an SAE, a Serious Adverse Event Form should be completed and reported to the Hospital Medical Ethics Committee and the IIT Project Management Office within 24 hours.

4.3 Assessment of Adverse Event Relatedness
> The investigator shall assess the potential relationship between the adverse event and the investigational intervention. The criteria for judging the relationship are as follows:
> ① Definitely not related: The AE has no reasonable possibility of being caused by the investigational intervention.
> ② Probably not related: Evidence suggests the event is more likely related to other factors (e.g., concomitant medication, concurrent illness), but a relationship to the intervention cannot be ruled out.
> ③ Possibly related: The event occurs in a reasonable time sequence following intervention administration, and could possibly be caused by the intervention. Cannot be ruled out if possibly caused by other factors (e.g., concomitant medication, concurrent illness). Withdrawal/rechallenge information unavailable or unclear.
> ④ Definitely related: The event type is a known reaction of the intervention, and cannot be explained by other reasons (e.g., concomitant medication, concurrent illness). The temporal relationship strongly suggests causality (e.g., response to withdrawal/rechallenge).
> ⑤ Not assessable: Insufficient information to make a judgment regarding causality. The investigator may change the causality assessment based on subsequent follow-up information and modify the corresponding AE/SAE report.

5. Data Collection and Management

5.1 Data Collection
> Electronic data recording.

5.2 Data Management
Use EXCEL for data entry, organization, and database establishment.

6. Statistical Analysis Plan

6.1 Sample Size Estimation
Sample size was determined using $N=2*[(Z_{\alpha/2}+Z_{\beta})\sigma/d]^2$ and statistical power analysis software G*Power 3.1. Referring to the effect size observed in studies like Dawson et al. (Cohen's d=0.632), with $\alpha=0.05$ and statistical power ($1-\beta$) equal to 80%, the estimated sample size per group should be 34. Considering a 10% clinical dropout rate, the sample size per group was adjusted to 38, totaling 114 participants across three groups.

Reference: Dawson J, et al. Vagus nerve stimulation paired with rehabilitation for upper limb motor function after ischaemic stroke (VNS-REHAB): a randomised, blinded, pivotal, device trial. Lancet. (2021) 397:1545–53.

6.2 Efficacy Analysis and Statistical Methods
Statistical analysis will be performed using R software. For demographic and baseline characteristics, continuous variables conforming to normal distribution will be expressed as mean ± standard deviation ($\bar{x} \pm s$) and compared between groups using independent samples t-test; if not normally distributed, they will be expressed as median and interquartile range (IQR) and analyzed using Mann-Whitney U test. Categorical variables will be compared using Chi-square test or Fisher's exact test based on sample size and expected frequencies.

For primary and secondary outcome measures (e.g., FMA-UE, FMA-LE, WMFT, BBS, MBI scores), Linear Mixed Effects Models (LMM) will be used to handle repeated measures data, implemented via the "nlme" package in R, to assess changes over time and differences between groups, accounting for correlations within subjects across multiple time points. Post-hoc subgroup analyses will explore differential efficacy of taVNS across different stroke types (ischemic vs. hemorrhagic) and lesion locations.

For safety indicators, normality tests will first be performed on Heart Rate Variability (HRV) and blood pressure data. If normally distributed, paired sample t-tests will be used to compare values before and during treatment; if not, Wilcoxon signed-rank test will be used. Comparison of adverse event incidence rates between groups will use Chi-square test or Fisher's exact test. $p < 0.05$ will be considered statistically significant.

6.3 Analysis Populations
Subjects who do not comply with the study protocol will be excluded from the analysis.

7. Research Related Ethics

7.1 Ethics Committee Review

> This protocol, the written informed consent form, and any materials directly related to subjects must be submitted to the Ethics Committee. The study may only formally commence after obtaining written approval from the Ethics Committee. The investigator must submit an annual study report to the Ethics Committee at least once a year. Upon study termination and/or completion, the investigator must notify the Ethics Committee in writing; the investigator must promptly report all changes occurring during the research work (such as revisions to the protocol and/or informed consent form) to the Ethics Committee and must not implement new modifications arbitrarily without prior approval from the Ethics Committee, unless changes are necessary to eliminate an apparent and immediate risk to subjects. In such cases, the Ethics Committee will be notified.

7.2 Informed Consent
The investigator must provide the subject or their legal representative with an easily understandable informed consent form approved by the Ethics Committee and give the subject or their legal representative sufficient time to consider participation in this study. No subject may be enrolled before obtaining their signed written informed consent.
During the subject's participation, all updated versions of the informed consent form and written information will be provided to the subject. The informed consent form shall be retained as an important document for the clinical trial for future reference.

8. Confidentiality Measures
The results of this research project may be published in medical journals, but we will keep the patient's information confidential as required by law. Unless required by relevant laws, the patient's personal information will not be disclosed. When necessary, government regulatory authorities, the hospital Ethics Committee, and their relevant personnel can access the patient's data according to regulations.

9. Quality Assurance Measures
(1) **Scientific Feasibility:** Both international and domestic literature, as well as our preliminary work, indicate that unilateral taVNS can improve upper limb motor dysfunction after stroke, and fNIRS has found that taVNS can activate the motor cortex in stroke patients. Based on this, this project plans to conduct a multicenter clinical trial of bilateral taVNS for treating upper and lower limb motor function in stroke hemiplegia, possessing a solid theoretical foundation.
(2) **Clinical Trial Conditions Met:** The leading unit, the Rehabilitation Medicine Department of the First Affiliated Hospital of Nanchang University, is a National Clinical Key Specialty, a Leading Medical Discipline in Jiangxi Province, and the initiating unit of the Jiangxi Provincial Rehabilitation Specialty Medical Alliance, admitting a large number of stroke patients with motor dysfunction annually, providing sufficient research subjects for this trial. The participating centers, The Rehabilitation Hospital Affiliated to Nanchang University and The First Affiliated Hospital of Gannan Medical University, are large comprehensive tertiary Grade A hospitals, whose Rehabilitation Medicine Departments also admit a large number of stroke patients each year. Furthermore, our department already possesses bilateral taVNS devices and fNIRS assessment equipment, meeting the necessary conditions for completing the

clinical trial.
(3) **Reasonable Research Team:** Our research group has long been committed to the rehabilitation of brain injury dysfunction using neuromodulation techniques. The project team involves multidisciplinary collaboration including Rehabilitation Medicine, Neuroelectrophysiology, and Imaging Department. The research team structure is reasonable, with clear division of labor and strong cooperation ability. Relevant members of the preliminary team possess the capability to complete the project, making it feasible in terms of the research team.
(4) **Solid Preliminary Work Foundation:** Under the leadership of Professor Feng Zhen, Vice President of the Chinese Rehabilitation Medicine Association, Chairman of the Rehabilitation Therapy Professional Committee of the Chinese Rehabilitation Medicine Association, and Vice Chairman of the Consciousness Disorder Rehabilitation Professional Committee of the Chinese Rehabilitation Medicine Association, the project applicant has long been engaged in clinical and basic research on brain injury rehabilitation. Currently, the applicant hosts 2 National Natural Science Foundation projects (1 Youth project, 1 Regional project), 3 provincial-level projects (2 Provincial Natural Science Foundation projects, 1 Provincial Clinical Research Cultivation Project), and has participated in multiple National Natural Science Foundation projects. He has published 15 papers as first author or corresponding author (including 8 SCI papers). Furthermore, the preliminary research achievements "Establishment and Application of Key Technologies for Coma Arousal using Peripheral Nerve Electrical Stimulation" and "Technological Innovation and Promotion of Neuromodulation for Chronic Disorders of Consciousness" won the second prize of the Jiangxi Provincial Science and Technology Progress Award in 2022 and the first prize of the Chinese Rehabilitation Medical Association Science and Technology Progress Award in 2023, respectively. Additionally, the project applicant and team members are proficient in the basic requirements of clinical research and possess a solid preliminary foundation.

10. Expected Schedule and Completion Date
This project is expected to be completed in 3 years. The overall schedule and progress are as follows:

[January 2025 - June 2025]{.underline}
Prepare and refine all pre-trial tasks, explain patient inclusion and exclusion criteria, conduct standardized training for scale administration, and provide homogeneous training for taVNS treatment to achieve standardized homogeneity across the three clinical centers. Additionally, complete ethics applications and approvals at the three clinical centers, and complete registration on the Chinese Clinical Trial Registry website.

[July 2025 - June 2026]{.underline}
Begin enrolling stroke patients first in the Rehabilitation Medicine Department of the First Affiliated Hospital of Nanchang University, administer taVNS treatment, monitor safety, and perform assessments including FMA-UE, FMA-LE, WMFT, Balance evaluation and training system, BBS, FAC, MBI, and fNIRS.

[July 2026 - December 2027]{.underline}
Simultaneously initiate trial enrollment in the Rehabilitation Medicine Departments of The Rehabilitation Hospital Affiliated to Nanchang University and The First Affiliated Hospital of Gannan Medical University, administer taVNS treatment, monitor safety, and perform assessments including FMA-UE, FMA-LE, WMFT, Balance evaluation and training system, BBS, FAC, MBI, and fNIRS.

[December 2027 - June 2028]{.underline}
Conduct supplementary clinical trials, data collection and processing, statistical analysis of experimental data, manuscript writing, and final reporting.

11. Forms of Research Output Dissemination
(1) Plan to publish 1-2 related research academic papers, including no less than 1 high-impact SCI journal paper, and actively apply for 1-2 scientific research achievements and patents.
(2) Summarize the clinical application protocol for bilateral taVNS, establish ⩾3 clinical application demonstration units, and promote and apply this technology within the province relying on the platform of the "Jiangxi Provincial Rehabilitation Specialty Medical Alliance".

12. References
[1] to [16] (References list remains unchanged in the translation as per the original Chinese numbers and formatting).
